# Supplementary material for: Diagnostic Accuracy of LDBIO-Toxo II IgG and IgM Western Blot in Suspected Seroconversion in Pregnancy: A Multicentre Study
Source: Pathogens. 2022 Jun 8;11(6):665. doi: 10.3390/pathogens11060665 (PMC9231380; doi:10.3390/pathogens11060665)
Supplement: Supplementary file 1 [file pathogens-11-00665-s001.zip › pathogens-1742323-supplementary.pdf]

Supplementary File S1: Head-to-Head Comparison Between WB IgM and Other Techniques

Methodology

This comparison was performed on a subset of samples on whom each individual technique was performed. The classification (seroconversion or false-positive) of each patient was kept.

For sensitivity analysis, all samples from seroconversion were considered positive. For specificity, all samples from false-positive were considered negative.

For all comparisons, equivocal tests were considered negative.

Confidence intervals were calculated using Wilson's score with continuity correction.

Test sensitivity and specificity were compared using the chi-squared test

In addition to the sample analysis, a head-to-head comparison between tests was performed on the samples to investigate whether a technique detected samples prior to the other one. Note that samples from the same patients, not including the head-to-head comparison, were not taken into consideration for determining the faster technique (except for the two exceptions detailed below for constantly negative tests).

If a technique failed to detect the patient constantly, it was also specified with the following exceptions:

- If a previous test on the same patient in the sample set but outside the comparison was positive with the test negative at the time of the comparison, then the patient was excluded on the faster/slower analysis and the constantly negative comment.
- If the following test on the same patient in the sample set but outside the comparison was positive, the test was considered slower but not constantly failing.

Results

WB IgM and Liaison IgM (Diasorin)

A total of 33 seroconversions and 49 false-negative patients were included, representing 78 positive samples and 116 negative samples.

	Western Blot IgM			Diasorin IgM		
	Positive	Equivocal	Negative	Positive	Equivocal	Negative
Seroconversions (*n* = 78)	65 (83.3%)	7 (9.0%)	6 (7.6%)	60 (76.9%)	3 (3.8%)	15 (19.2%)
False positive (*n* = 116)	7 (6.0%)	34 (29.3%)	75 (64.7%)	45 (38.8%)	16 (13.8%)	55 (47.4%)
Total	72	41	81	105	19	70

WB IgM sensitivity and specificity were 83.3% (95CI 72.8–90.5%) and 94.0% (95CI 87.5–97.3%), respectively.

Diasorin IgM sensitivity and specificity were 76.9% (95CI 65.7–85.4%) and 61.2% (95CI 51.7–70.0%), respectively.

There was no statistical difference for sensitivity ($p = 0.32$), but WB had better specificity ($p < 0.001$).

Regarding per-patient analysis, WB was faster for four patients, Diasorin for five and both tests were detected simultaneously 24 times. Two patients remained constantly negative for Diasorin IgM.

WB IgM and Architect IgM (Abbott)

A total of 44 seroconversions and 17 false-negative patients were included, representing 112 positive samples and 36 negative samples.

	Western Blot IgM			Architect IgM		
	Positive	Equivocal	Negative	Positive	Equivocal	Negative
Seroconversions (*n* = 112)	87 (77.7%)	7 (6.3%)	18 (16.1%)	83 (74.1%)	0 (0%)	29 (25.9%)
False positive (*n* = 36)	7 (19.4%)	13 (8.3%)	16 (72.2%)	28 (77.8%)	2 (2.8%)	6 (19.4%)
Total	94	20	34	111	2	35

WB IgM sensitivity and specificity were 77.7% (95CI 68.6–84.8%) and 80.6% (95CI 63.4–91.2%), respectively.

Architect IgM sensitivity and specificity were 74.1% (95CI 64.8–81.7%) and 22.2% (95CI 10.7–39.6%), respectively.

There was no statistical difference for sensitivity ($p = 0.53$), but WB had better specificity ($p < 0.001$).

Regarding per-patient analysis, WB was faster for three patients, Architect for one and both tests were detected simultaneously 40 times. Two patients remained constantly negative for Architect IgM.

WB IgM and Vidas IgM (BioMérieux)

A total of 20 seroconversions and 17 false-negative patients were included, representing 48 positive samples and 38 negative samples.

	Western Blot IgM			Vidas IgM		
	Positive	Equivocal	Negative	Positive	Equivocal	Negative
Seroconversions (*n* = 48)	43 (89.6%)	5 (10.4%)	0 (0%)	43 (89.6%)	1 (2.1%)	4 (8.3%)
False positive (*n* = 38)	8 (21.1%)	3 (7.9%)	27 (71.1%)	26 (68.4)	2 (5.3%)	10 (26.3%)
Total	51	8	27	69	3	14

WB IgM sensitivity and specificity were 89.6% (95CI 76.5–96.1%) and 78.9% (95CI 62.2–89.9%), respectively.

Vidas IgM sensitivity and specificity were 89.6% (95CI 76.5–96.1%) and 31.6% (95CI 18.0–488%), respectively.

There was no statistical difference for sensitivity ($p = 1$), but WB had better specificity ($p < 0.001$).

Regarding per-patient analysis, WB was faster for three patients, Vidas IgM for two and both tests were detected simultaneously 15 times. No patients remained constantly negative for any test.

WB IgM and Platelia IgM (Bio-Rad)

A total of 15 seroconversions and 0 false-negative patients were included, representing 34 positive samples and 0 negative samples.

	Western Blot IgM			Platelia IgM		
	Positive	Equivocal	Negative	Positive	Equivocal	Negative
Seroconversions (*n* = 34)	30 (88.2%)	4 (11.7%)	0 (0%)	34 (100%)	0 (0%)	0 (0%)
False positive (*n* = 0)	Not applicable					
Total	30	4	0	34	0	0

WB IgM sensitivity was 88.2% (95CI 66.5–93.0%).

Platelia IgM sensitivity was 100% (95CI 87.3–99.7%).

Platelia IgM had better sensitivity ($p = 0.0393$).

Regarding per-patient analysis, WB was faster for no patients, Platelia IgM for four and both tests were detected simultaneously 11 times. No patients remained constantly negative for any test.

WB IgM and ISAGA Bio Mérieux

A total of 93 seroconversions and 61 false-negative patients were included, representing 148 positive samples and 110 negative samples.

	Western Blot IgM			ISAGA		
	Positive	Equivocal	Negative	Positive	Equivocal	Negative
Seroconversions (*n* = 148)	122 (82.4%)	16 (10.8%)	10 (6.8%)	130 (87.8%)	6 (4.1%)	12 (8.1%)
False positive (*n* = 110)	10 (9.1%)	26 (23.6%)	74 (67.3%)	53 (48.2%)	6 (5.5%)	51 (46.4%)
Total	132	42	84	183	12	63

WB IgM sensitivity and specificity were 82.4% (95CI 75.1–88.0%) and 90.9% (95CI 83.5–95.3%), respectively.

ISAGA IgM sensitivity and specificity were 87.8% (95CI 81.2–92.4%) and 51.8% (95CI 42.1–61.4%), respectively.

There was no statistical difference for sensitivity ($p = 0.19$), but WB had better specificity ($p < 0.001$).

Regarding per-patient analysis, WB was faster for 2 patients, ISAGA for 11 and both tests were detected simultaneously 68 times. Two patients remained constantly negative for western blot and two others for ISAGA. Two patients were removed from comparison; one was WB negative and

ISAGA positive but was WB positive on a previous sample (ISAGA not performed) and the second one was negative for both tests but positive on the following sample for WB (ISAGA not performed).

Discussion

Overall, the western blot IgM showed a sensitivity close to the other IgM techniques, with the exception of Platelia IgM, which showed greater sensitivity. However, as the samples were selected according to their IgG and IgM values and not WB, this creates a bias against the western blot.

On the opposite, the western blot showed far greater specificity than other techniques. As samples were selected when at least one IgM technique gave false-negative results, this is not surprising, as it creates a bias in favour of the western blot. However, it shows that WB can be used to rule out most false negatives for all techniques in the study.

It was not possible to compare other techniques, so it was not possible to estimate whether one technique is more reliable than another in our study.
